# Molecular Mechanism for the Regulation of ABA Homeostasis During Plant Development and Stress Responses

**DOI:** 10.3390/ijms19113643

**Published:** 2018-11-19

**Authors:** Yanlin Ma, Jing Cao, Jiahan He, Qiaoqiao Chen, Xufeng Li, Yi Yang

**Affiliations:** Key Laboratory of Bio-Resources and Eco-Environment of Ministry of Education, College of Life Sciences, Sichuan University, Chengdu 610065, China; maylok@163.com (Y.M.); Caojing94@163.com (J.C.); SCU_hjh@163.com (J.H.); chenqqg7@sina.com (Q.C.); lixufeng0507@gmail.com (X.L.)

**Keywords:** ABA production, ABA inactivation, ABA transporter, ABA homoeostasis, transcriptional regulation, stress response

## Abstract

The plant hormone abscisic acid (ABA) play essential roles in numerous physiological processes such as seed dormancy, seed germination, seeding growth and responses to biotic and abiotic stresses. Such biological processes are tightly controlled by a complicated regulatory network including ABA homoeostasis, signal transduction as well as cross-talking among other signaling pathways. It is known that ABA homoeostasis modulated by its production, inactivation, and transport pathways is considered to be of great importance for plant development and stress responses. Most of the enzymes and transporters involved in ABA homoeostasis have been largely characterized and they all work synergistically to maintain ABA level in plants. Increasing evidence have suggested that transcriptional regulation of the genes involved in either ABA production or ABA inactivation plays vital roles in ABA homoeostasis. In addition to transcription factors, such progress is also regulated by microRNAs and newly characterized root to shoot mobile peptide-receptor like kinase (RLKs) mediated long-distance signal transduction. Thus, ABA contents are always kept in a dynamic balance. In this review, we survey recent research on ABA production, inactivation and transport pathways, and summarize some latest findings about the mechanisms that regulate ABA homoeostasis.

## 1. Introduction

The hormone abscisic acid (ABA) was first isolated from plants including cotton and potato in 1960s [1]. Increasing evidences show that ABA plays key role in the whole life of plants [2]. ABA regulates not only the growth and development of plants but also the responses to biotic and abiotic stresses [3,4,5]. One of the most study on ABA action in response to adverse environmental conditions is the modulation of stomatal movement. When suffered from drought stress, plants elevate ABA levels and activate ABA receptors by forming ABA-receptor complexes [6]. Subsequently, these ABA-receptor complexes interact with PP2Cs (protein phosphatase 2Cs) and relieve the inhibitory effect of PP2Cs on downstream kinases such as OST1 (Open stomata 1) and CDPKs (calcium-dependent protein kinases), these protein kinases phosphorylate the slow anion channel-associated 1 (SLAC1) and activate its channel activity [7]. The activated SLAC1 mediates anion efflux, which triggers stomatal closure and confers plant drought resistance [8,9]. Cellular ABA level fluctuates continuously, enabling plants to meet the needs of growth and development as well as cope with the changing environmental conditions. ABA levels increase rapidly during seed maturation or when plants are challenged by stress conditions, while ABA levels decrease quickly under some conditions such as rehydration and during seed germination, suggesting that regulation of ABA homoeostasis is indispensable for plant development and adaptive responses to various stress conditions [10,11]. The homoeostasis of ABA in plants is regulated by its production, inactivation and transport pathways. A series of cellular components including enzymes, transporters as well as other molecules have been identified to play a role in these pathways and all of them in these pathways work synergistically to modulate ABA homoeostasis [12]. Revealing the roles of these components in ABA homoeostasis is the basis for investigating ABA-mediated signal transduction and various physiological processes. Moreover, such research work is crucial to develop new genetically modified crops with high-yielding and enhanced resistance to adverse stresses. In this review, we discuss recent findings on ABA production, inactivation and transport as well as regulatory mechanisms in the maintenance of ABA homoeostasis in plants.

## 2. ABA Biosynthesis and Degradation Pathway

**Figure 1:**
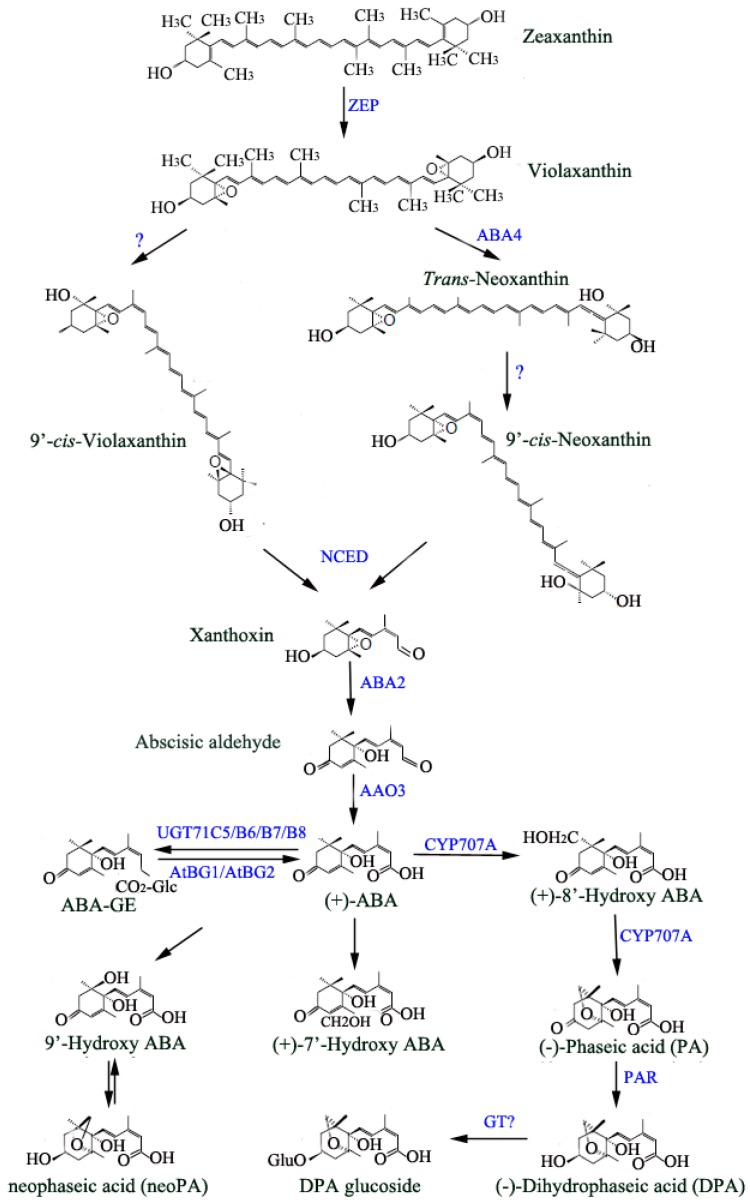
Overall scheme for abscisic acid (ABA) biosynthetic and catabolic pathways in Arabidopsis. ABA de novo biosynthesis is catalyzed by a series of enzymes including ZEP, ABA4, NCED, ABA2 and AAO3, while ABA degradation is mainly catalyzed by CYP707A and the products are PA and DPA. The UDP-glucosyltransferase UGT71C5/B6/B7/B8 modify ABA to ABA-GE, while the glycosyl hydrolase AtBG1/2 convert ABA-GE to ABA. Enzymes (blue) and compound names (black) discussed in the text are shown in the figure. Enzymes are denoted next to the catalytic steps. The main ABA metabolic pathways are shown with black arrows.

Since ABA was discovered, its biosynthetic pathway has been widely studied by characterizing a series of mutants at almost all enzymatic steps in Arabidopsis and other plants [13]. It is generally believed that ABA is synthesized de novo mostly through carotenoids pathway in higher plants [14]. ABA de novo biosynthesis is mediated by multi-step enzymatic reactions. The first step of ABA biosynthesis is starting from zeaxanthin, a kind of carotenoids, the reaction is catalyzed by zeaxanthin epoxidase (ZEP) to produce violaxanthin, the following reaction is catalyzed by ABA-deficient 4 (ABA4), the product, trans-neoxanthin, is subsequently converted to 9-*cis*-neoxanthin by an unclear mechanism, formation of 9-*cis*-violaxanthin is also converted from violaxanthin by unknown reaction. Both 9-*cis*-neoxanthin and 9-*cis*-violaxanthin serve the substrates of 9-*cis*-epoxycarotenoid dioxygenases (NCEDs), xanthoxin is produced in this step and relocalized from plastid to cytoplasm. The final two steps of ABA biosynthesis occur in the cytoplasm rather than in plastid. In the cytoplasm, xanthoxin is converted to ABA-aldehyde by ABA-deficient 2 (ABA2), which belongs to short-chain dehydrogenase /reductase (SDR) family. The last step of ABA biosynthesis is mediated by ABA-aldehyde oxidase 3 (AAO3), which catalyzes ABA-aldehyde to produce ABA [15] (Figure 1). 

Among the enzymes in the ABA biosynthetic pathway, NCEDs function as the key rate-limiting components [16]. Since VP14 was cloned as the first NCED-encoding gene in maize [17], extensive studies on NCEDs promoted understanding of the regulatory network of ABA synthesis. So far, there are five NCEDs (AtNCED2, AtNCED3, AtNCED5, AtNCED6, AtNCED9) which are localized in plastid, that are found to be responsive to ABA synthesis in Arabidopsis [18]. The analysis of gene expression showed that both *NCED2* and *NCED3* had high expression levels in roots and leaves but low levels remained during seed development, whereas the expressions of *NCED5*, *NCED6* and *NCED9* raised during late maturation stages [18]. This result suggests a differential contribution of NCEDs in ABA production during plant growth and development. Almost all of *NCEDs* that have a potential function in seeds due to their expression can be positively regulated by the transcription factor MYB96 (MYB domain protein 96), which was considered to play a role in seed dormancy [19]. Among them, *NCED6* and *NCED9* are expressed in the endosperm and embryo of developing seeds, respectively, and both of their products are major enzymes responsive to ABA biosynthesis in seeds. The *nced6nced9* double mutants exhibit lower ABA level in seeds and markedly reduced dormancy compared to wild type plants and single mutants, suggesting the functional redundancy of NCED6 and NCED9 in regulating seed dormancy [20,21].

**Figure 2:**
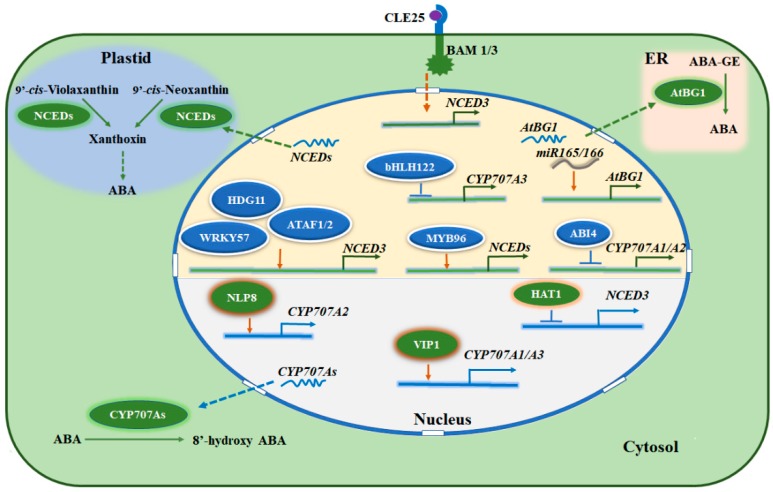
Transcriptional regulation network of the genes involved in ABA production and inactivation. Transcription factors in upper half of the nucleus positively contribute ABA content while transcription factors in the lower half play the opposite role. In nucleus, green and blue lines show promoter region of the related genes, arrow heads (brown) and end lines (blue) indicate positive and negative regulation, respectively. Green and blue dotted arrows represent nuclear export of the mRNA involved in ABA production and inactivation, respectively. Brown dotted arrow from cytosol to nucleus indicates an unknown pathway. In cytosol, green arrow indicates ABA catabolic pathway. In plastid, green arrows and dotted arrows represent ABA one-step and multi-step biosynthetic pathway, respectively. In ER, green arrow indicates ABA glycosylation pathway. ER, endoplasmic Reticulum.

In addition to its role in plant growth and development, ABA plays a crucial role in response to abiotic stresses, especially when facing drought stress, plants elevate cellular ABA level quickly. In Arabidopsis, NCED3 is considered as a dominating contributor to ABA production under water deficit, while other NCEDs, as well as other enzymes within ABA synthesis pathway such as ZEP and AAO3, play a relatively minor role [18,22,23]. The action of ABA on stomatal movement is a central regulatory component in drought stress response. It was recently reported that *NCED3* was strongly induced by sulfate, a potential chemical signal of drought, which increases ABA level and promotes stomatal closure [24]. More recently, a root-derived peptide, CLE25 (Clavata3/ESR-Related 25), was identified as a key regulator of *NCED3* expression in leaves (Figure 2). *CLE25* is mainly expressed in vascular tissue of roots, its transcripts are dramatically induced when plants have suffered from drought stress. CLE25 moves from root to leaves and associates with two receptor-like protein kinases (RLKs), BAM1 (Barely Any Meristem 1) and BAM3, thus elevating ABA level by inducing *NCED3* expression [25]. Genetic evidences show that low ABA level was detected in both *cle25* and *bam1/3* mutants which, in turn, were more sensitive to dehydration stress compared to wild type plants. Grafting experiments indicate that BAM1/3 function in leaves rather than roots under drought stress. These findings suggest that peptide-RLK mediated long-distance signaling plays a vital role in the regulation of ABA homeostasis [25]. Additionally, *NCED3* transcripts are also induced rapidly when leaf turgor decreases [26]. Other stresses such as cold and high salt significantly induce *NCED3* and *NCED5* under the control of an mRNA decapping complex [27]. Recently, the class II HD-ZIP transcription factors, HAT1 and HAT3, were identified as new regulators of ABA homeostasis. Plants overexpressing *HAT1* exhibit ABA-insensitive phenotype and are less tolerant to drought stress, while *Hat1Hat3* double mutants show enhanced tolerance to drought stress compared with wild type plants. Electrophoresis mobility shift assays (EMSA) and chromatin immunoprecipitation (ChIP)-qPCR assays prove that HAT1 is able to bind *NCED3* promoter region, further result shows HAT1 represses *NCED3* expression, suggesting that HAT1 and HAT3 negatively regulate ABA homeostasis through ABA biosynthetic pathway [28]. Besides, *NCED3* expression is also modulated by other transcription factors such as WRKY57 (WRKY DNA-binding protein 57), BDG1 (9-*cis* epoxycarotenoid dioxygenase defective 1) and ANAC2 (Arabidopsis NAC domain containing protein 2) in Arabidopsis, thereby conferring improved tolerance to drought or heat stress [29,30,31]. These results together demonstrate that NCEDs, the key enzymes in ABA production, are not only indispensable during plant growth and development but also play an essential role in coping with multiple stress conditions.

Compared to its biosynthesis, ABA degradation seems to be less complicated. There is ample proof to show that ABA is degraded by hydroxylation which takes place at three various methyl groups (C-7′, C-8′ and C-9′), hydroxylated ABA is voluntarily isomerized to phaseic acid (PA), then it is further catalyzed to dihydrophaseic acid (DPA), both PA and DPA are the major ABA catabolites with weak and no bioactivity, respectively [32,33]. ABA 8′-hydroxylation is mediated via cytochrome P450 (CYP) 707A subfamily monooxygenases (CYP707A1-4), which is considered as a primary catabolism pathway in Arabidopsis [34]. The expressions of four *CYP707As* are observed in multiple tissues although their relative abundances are distinct. Previous results showed that *CYP707A1* mRNA accumulated during mid-maturation and decreased rapidly during late maturation of seeds. Moreover, high expression levels of *CYP707A1* and *CYP707A3* were detected in silique, suggesting that both play roles in seed development [35]. CYP707A2 is mainly found in dry seed, which is consistent with its function in ABA degradation at the end of seed development and seed germination. Besides, genetic evidences show that *cyp707a1cyp707a2* double mutants accumulate high level of ABA and exhibit pronounced dormancy compared with single mutants and wild type plants, indicating that both CYP707A1 and CYP707A2 play negative roles during seed dormancy [36]. Recently, the transcription factor ABI4 was identified as a negative regulator in the expressions of *CYP707A1* and *CYP707A2* in the manner of directly binding to the promoters of these two genes during seed dormancy [37] (Figure 2). In addition, *CYP707A2* is also reported to be involved in nitrate stimulated seed germination and its expression is positively modulated by NLP8 (NIN-like protein 8), a RWP-RK domain transcription factor, in the presence of nitrate [38], suggesting that nitrate signaling also controls ABA homoeostasis (Figure 2). Although there is no detailed report about the function of *CYP707A4*, a relatively higher expression level was detected in flowers at normal growth conditions, indicating a potential role of CYP707A4 in ABA catabolism during flowering [39]. These findings reveal that CYP707As play essential and different roles during plant growth and development. When plants are challenged by adverse conditions such as drought stress, cellular ABA level rises rapidly due to the induction of genes involved in ABA production. It was recently reported that VIP1 (VirE2-interacting protein 1), a bZIP transcription factor, plays a role in ABA-mediated osmosensory signaling. The transcripts of *VIP1* accumulate instantly during rehydration, which correlates with the decreasing content of ABA [40]. Further research indicates that VIP1 directly binds to the promoter regions of *CYP707A1* and *CYP707A3* to positively control the expression of these two genes, leading to a reduced ABA content during rehydration [39]. Recent research shows that the MADS-box transcription factor SHORT VEGETATATIVE PHASE (SVP), functions as a positive regulator of drought resistance by binding the CArG Motifs in the promoters of *CYP707A1*, *CYP707A3* and *AtBG1*, causing decreased expression of *CYP707A1* and *CYP707A3*, but enhanced expression of *AtBG1* in Arabidopsis leaves and substantially increases cellular ABA levels [41]. Another transcription factor, known as bHLH122, which belongs to the basic helix-loop-helix (bHLH) family, was identified to play a central role in multiple stress responses such as drought, salt and osmotic stresses [40]. bHLH122 notably reduces the expression of *CYP707A3* by binding to the G-box/E-box at its promoter region (Figure 2). As a consequence, ABA is accumulated so that it improves the tolerance of plants to stress conditions [40]. These results strongly demonstrate that ABA levels are synergistically modulated through both biosynthetic and catabolic pathways and the transcriptional regulation of the related genes is an important process for controlling ABA homoeostasis in plants (Figure 1).

## 3. Reversible Glycosylation of ABA

Apart from de novo biosynthesis and catabolism pathways, the homoeostasis of ABA is also regulated by reversible glycosylation mediated by the ABA-UGTs (uridine diphosphate glucosyltransferases), a group of plant glucosyltransferase subfamily [42,43,44,45]. Previous studies have shown that three functionally redundant enzymes, known as UGT71B6/7/8, conjugate ABA with glucose to generate ABA glucosyl ester (ABA-GE), which lacks biological activity and is considered as a transported form, which can be transported from cytoplasm to the vacuole and endoplasmic reticulum (ER) in Arabidopsis [46]. Genetic evidences revealed that the three *UGT71Bs* act as important performers of ABA homoeostasis in regulating plant growth and the response to various abiotic stresses including drought, osmotic and salt stress [46]. Although no distinguishable phonotypes were observed for wild type, *UGT71B6* overexpression lines and *UGT71B6* single mutants during seed germination, plants overexpressing *UGT71B6* exhibited less sensitivity to exogenous ABA compared with wild type plants during seeding growth, suggesting that relative less contribution of UGT71B6 on ABA inactivation under normal condition and the level of ABA in *UGT71B6* overexpressing plants may be complemented by other enzymes involved in ABA production. However, compared to their single mutants, the *UGT71B6/7/8* RNAi triple mutants showed multiple growth defects with smaller rosette leaves, shorter roots and pale-green leaves [46]. Besides, both seed germination and seeding growth of the triple mutants are suppressed by exogenously applied ABA compared with those of wild type and single mutants, and the triple mutants are also more tolerant to osmotic and drought stresses [46,47]. Recently, a new glucosyltransferase, UGT71C5, was characterized as a predominant contributor in the regulation of ABA homoeostasis. Biochemical results implied that *UGT71C5* single mutant accumulated more ABA and less ABA-GE, whereas low level of ABA and high level of ABA-GE were detected in its overexpression lines compared with wild type plants [48]. Indeed, ABA content caused by the UGT71C5 action is closely related to the gene expression changes of ABA signal pathway, suggesting that UGT71C5 plays a key role in ABA homoeostasis and modulation of ABA signal transduction. Additionally, compared to minor contribution of UGT71B6 in seed germination, *UGT71C5* plants show a pronounced delay in seed germination. UGT71C5 was also proven to function as a vital regulator in response to dehydration stress, plants lacking functional UGT71C5 exhibited enhanced resistance to drought stress, while *UGT71C5* overexpression lines were more sensitive to water deficit compared with wild type plants [48,49,50]. 

Glucose conjugation of ABA not only affects its bioactivity but also changes its subcellular localization [43,51]. ABA-GE catalyzed by UGTs can be transported and stored in ER or vacuole. ABA-GE is also hydrolyzed to ABA via one-step enzymatic reaction when plants are challenged with drought stress, suggesting that reversible conversion between ABA-GE and ABA is a rapid and crucial process for the maintenance of ABA homoeostasis. Until now, three glucosidases, AtBG1, AtBG2 and BGLU10, were characterized as important enzymes responsive to the conversion of ABA-GE to ABA in Arabidopsis. AtBG1 is located in ER while AtBG2 and BGLU10 are located in vacuoles [30]. Similar to other enzymes involved in ABA production and inactivation, functional deficiency of ABA-glucosidases also alters intracellular ABA level, thus affecting plant growth, development and the responses to adverse environmental conditions. It was previously reported that very low level of ABA remained in *ATBG1* plants with dwarf and yellow leaf phenotype, which was rescued by the application of exogenous ABA. Moreover, AtBG1 knockout plants are also sensitive to drought stress [18]. Interestingly, water deficiency dramatically enhances the enzymatic activity of AtBG1 by inducing its polymerization, implying that high molecular weight form of AtBG1 may play an important role in rapid ABA production under stress conditions [52]. On the contrary, AtBG2 is a relatively high molecular weight protein and has a low-level protein amount under normal conditions but accumulates rapidly via unclear mechanism when plants are facing challenge of dehydration [30], which indicates different regulatory mechanisms between AtBG1 and AtBG2 action on ABA homoeostasis and that a dynamic balance of AtBG protein amounts is essential for the adaptation to stress. Recently, BGLU10 was characterized as a new ABA-GE glucosidase, similar phenotypes were observed in *BGLU10* overexpressing and its loss-of-function plants, which was comparable to that of *AtBG2* [30]. Functional redundancy of AtBG2 and BGLU10 may be the answer to the question about why phenotypes of *atbg1* mutants are more obvious than *ATBG2* plants under stress conditions. These findings suggest that ER and vacuole, two important ABA pools, are indispensable for the rapid production of ABA in response to stress conditions and plants possess multiple strategies for the maintenance of ABA homoeostasis to cope with the altering environmental conditions in order to survive. 

## 4. ABA Transporters

**Figure 3:**
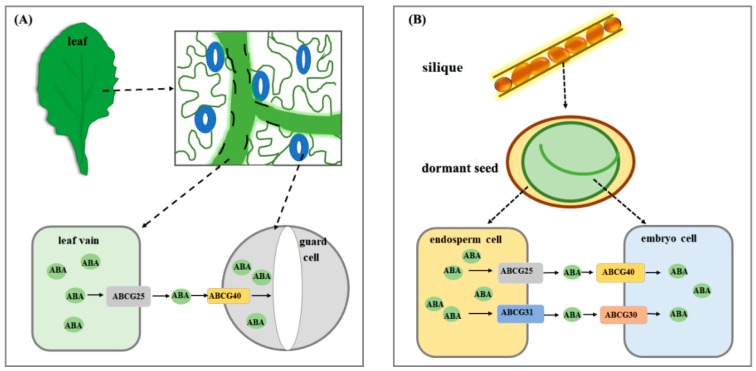
ABA transport system in leaf and dormant seed. (**A**) when plants are suffered from drought, ABCG25 transports ABA from leaf vein to the apoplastic area around guard cells, and subsequently ABA is taken up into guard cells by ABCG40, thus causing stomatal closure. (**B**) during seed dormancy, ABCG25 and ABCG31 mediate ABA efflux from endosperm cells, ABCG30 and ABCG40 transport ABA from extracellular space to embryo cells, which maintains seed dormancy. Solid arrows indicate the direction of ABCG-mediated ABA transportation. Dotted arrows show local enlarged view of specific cells, tissues and organs.

When the roots of plants are placed in a solution containing ABA, an increased ABA content is detected in leaves [53], implying that ABA can be transported from root to shoot. A previous point of view supposed that the root was only place for ABA synthesis and ABA was transported over a long distance to leaves [53]. The recent discovery of several ABA transporters strongly supports the idea that ABA is indeed transported by specific transporters and that it is transported in whole plant. The Arabidopsis ABCG25 (*Arabidopsis thaliana* ATP-binding cassata G25), belonging to ATP-binding cassette (ABC) transporter family, was identified as an ABA transporter. High expression level of *ABCG25* was observed in vascular tissues of both shoots and roots and ABCG25 was located in plasma membrane [54]. Biochemical experiments show that ABA is transported by ABCG25 and the transport activity is enhanced by the presence of ATP. Genetic evidences indicate that overexpression of *ABCG25* reduces the inhibition of ABA-mediated post-germinative growth and leads to a relative weak transpiration [54]. Because ABCG25 is not located in guard cell plasma membrane, the fact that guard cells of *ABCG25* overexpressing lines accumulate more ABA than wild type plants demonstrate that ABCG25 is an exporter that transports ABA into the apoplastic area around the guard cells [54]. These results raise the question that whether one or more guard cell plasma membrane-located transporters mediate the import of ABA to guard cells. This assumption was proved by the characterization of another ABA transporter, ABCG40, which is localized at the plasma membrane of guard cells and functions as an ABA importer. Plants lacking functional ABCG40 exhibit less sensitivity to ABA, including reduced expression of ABA-responsive genes and impaired stomatal closure [55]. It seems that the contribution of ABCG40 in guard cell ABA content is more obvious than ABCG25 as *ABCG40* mutants are more fragile under drought condition compared with those of *ABCG25* mutants and wild type plants. However, there is no doubt that ABCG25 and ABCG40 synergistically modulate the ABA content of guard cells and both of them play essential roles in ABA homoeostasis (Figure 3A). This kind of cooperative working-model among different ABCGs is supported by recent studies. The movement of ABA from endosperm to embryo is directed by four ABCG transporters in Arabidopsis (Figure 3B): Endosperm cell located ABCG25 and ABCG31 work as ABA exporters and mediate ABA efflux from the endosperm, while embryo cell located ABCG30 and ABCG40 import ABA into the embryo. All of their loss-of-function mutants show similar phenotype with less sensitivity to exogenous ABA compared with wild type plants during seed germination, suggesting that ABA homoeostasis is regulated by specific transporter genes expressed in different tissues during embryonic growth [56,57]. Another type of gene, named *ABCG22*, is induced by ABA and plants lacking functional ABCG22 show decreased stomatal closure under the conditions of low air humidity, elevated CO2 or exogenously applied ABA, indicating its potential role in the regulation of ABA homoeostasis although its ABA transporter activity still needs to be investigated [58]. In addition to ABC transporters, other types of transporters are also reported to possess ABA transport activity such as AIT1/NRT1.2 (ABA-importing transporter 1/nitrate transporter 1.2) and DTX50 (detoxification efflux carrier 50). AIT1/NRT1.2, which belongs to nitrate transporter (NRT) family and is localized at plasma membrane [59]. *AIT1* is expressed in vascular tissues and its loss-of-function mutants exhibit less sensitivity to ABA during seed germination and seeding growth, whereas *AIT1* overexpressing plants are hypersensitive to exogenously applied ABA compared with wild type plants [59]. DTX50, a member of Detoxification Efflux Carriers (DTX)/Multidrug and Toxic Compound Extrusion (MATE) transporter family, was identified as an ABA efflux transporter and played a key role in ABA-inhibited seeding growth and the response to dehydration stress. The seedlings of *dtx50* mutants show pronounced growth retardation and the adult plants are more tolerant to drought stress compared with wild type plants [60]. These findings support the notion that transporter-mediated ABA efflux and influx across the plasma membrane, as important as ABA production and inactivation, is indispensable for the control of ABA homoeostasis in plants and the ABA transporters are also crucial regulatory components in ABA modulated plant growth, development and the response to stress conditions.

## 5. Modulation of ABA Homoeostasis

**Figure 4:**
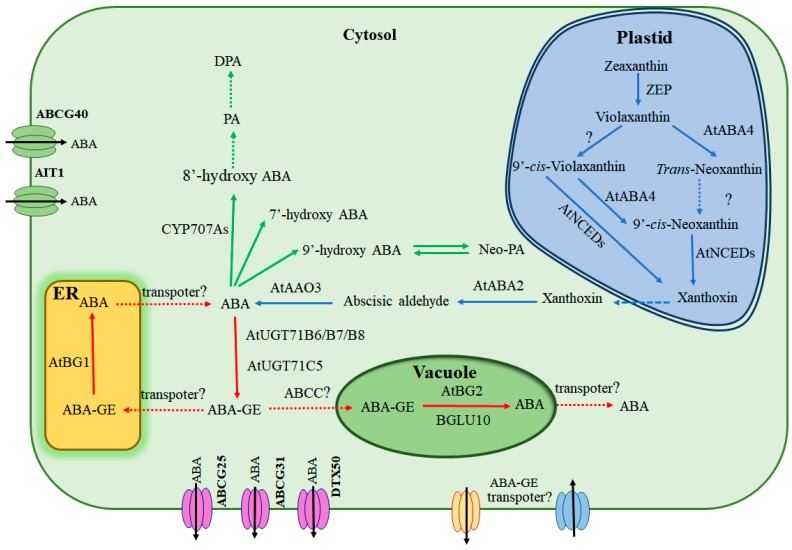
Regulatory network of ABA homoeostasis. The pathways which have been already reported are shown with solid arrows, while those unclear pathways are depicted by dotted arrows. The blue arrows represent de novo ABA biosynthetic pathway, green lines represent ABA catabolic pathway, red arrows represent reversible glycosylation of ABA, and black arrows indicate the direction of ABA transporters. Plasma membrane-located ABA transporters are pictured by different colors (green for ABA influx transporters and purple for ABA efflux transporters). Putative ABA and ABA-GE transporters at plasma membrane and various subcellular organelles are labeled by question marks.

The phrase “ABA homoeostasis” refers to the trend of plants to self-regulate and maintain their internal ABA content in a stable state. Multiple pathways maintain ABA homoeostasis, because there is a close connection between ABA production and inactivation pathways due to the complementation of deficiency of one pathway by other pathways. Either overexpression of *NCED3* or *AtBG2* can rescue the ABA deficient phenotype in *AtBG1* mutant [51]. On the other hand, increased transcript abundances of *CYP707As* as well as other ABA-responsive genes, were also detected both in UGT71B6/7/8 knockdown and UGT71C5 knockout plants, respectively [48,49]. Moreover, this complementary phenomenon was observed in plants that lack of functional CYP707As, whose ABA-GE level was much higher than the wild type plants [61], suggesting a possibility of the contribution by glucosyltransferases such as UGT71C5. A similar regulatory mechanism may be also existent during the conversion between ABA and ABA-GE. Because of less contribution of UGT71B6 action on seed germination, it is likely that the loss of ABA production in *UGT71B6* overexpressing plants may be compensated through the ABA-GE hydrolysis and ABA de novo biosynthesis mediated by AtBGs and NCEDs, respectively [47]. ABA content is tightly controlled by plants as the expressions of all *CYP707As* increase gradually during water deficit [35]. Furthermore, the mRNA amount of *CYP707A1* dramatically increased after rehydration, reached the peak only within one hour and then decreased quickly, the expressions of other *CYP707As* are opposite to *NCED3* and have a similar expression pattern during rehydration [62], suggesting that ABA homoeostasis is also regulated by feedback mechanisms, which balances plant growth and stress response. The above regulatory modes of ABA homoeostasis are mainly regulated at the transcriptional level. Indeed, several transcription factors have been recently identified as positive or negative regulators to control the expression of the genes involved in ABA biosynthesis and catabolism pathways [39,40,63,64,65]. Recently, non-coding RNAs were also found to be involved in ABA homoeostasis. Two microRNAs (miRs), miR165 and miR166, modulate plant development and the responses to abiotic stresses by directly controlling the expression of *AtBG1* as well as other ABA-responsive genes (Figure 2) [65]. Besides, it was previously reported that drought stress strongly induces the mRNA of *AAO3*, but the protein level and enzymatic activity of AAO3 are not altered [22,66]. AtBG1 can enhance its activity by forming a high molecular complex under water deficit condition, whereas AtBG2 comes to be a high molecular weight form and the protein level is very low under normal condition. Interestingly, the amount of AtBG2 complex dramatically increases under drought stress, implying that the post-translational regulation of these enzymes also plays a role in ABA balance (Figure 2) [51,52]. Additionally, ABA balance is also regulated by the transcription factor NLP8 and nitrate transporter NRT1.2, which were considered as the components of nitrate signal pathways [59,65]. Moreover, nitrate can dramatically induce the expression of *AtBG1* to increase ABA level in root tips [27]. These results strongly indicate that nitrate signal regulates ABA homoeostasis through multiple pathways including ABA production, catabolism and transport. Until now, the influence of ABA transport on other ABA homoeostasis pathways is still to be largely clarified, but it is possible that the altered expressions of those transporter genes may also affect the other ABA-related genes as DTX50 loss-of-function mutants accumulate more ABA and increase the expression of ABA-responsive genes such as *ABF1* [60]. These findings prove that a complicated and tightly regulatory network controls ABA homoeostasis in plants, including all the components in ABA production, inactivation and transport pathways (Figure 4).

## 6. Conclusions 

In higher plants, ABA homoeostasis is maintained by the balance of its biosynthesis, catabolism, reversible glycosylation and transport pathways. All of the components involved in ABA homoeostasis make up a large and complex regulatory network and they all function cooperatively to modulate ABA levels. Compared to ABA de novo biosynthesis and catabolism, the reversible glycosylation of ABA quickly regulates ABA levels by one step reaction, suggesting that both UGT-mediated glycosylation of ABA and BG-mediated hydrolyzation of ABA-GE are critical to the maintenance of ABA homoeostasis, especially under the stress conditions such as water deficit. ABA is compartmented in different regions of the cell including cytosol, ER and vacuole, suggesting that ABA-mediated signal transduction depends on the local ABA levels instead of the whole cellular ABA concentration, this notion is supported by the discovery of the three different ABA receptors localized in different organelles [67,68,69]. As a signal molecule in plants, ABA is transported from one region to another, the “region” includes different tissues, cells and organelles. Several ABA transporters were characterized during recent years and they were all localized at plasma membrane to control the intracellular and extracellular concentration of ABA. However, specific ABA or ABA-GE transporters involved in ABA transport across different organelles have not been reported yet. Still, it is possible that specific ABA transporters have such a role due to the studies about the vacuole-located ABCC-type ABC transporter from Arabidopsis, which possesses the activity of ABA-GE transport [70]. Thus, identification of new ABA or ABA-GE transporters may be an important research line, which will help us understand the regulatory mechanism of ABA homoeostasis more comprehensively. In summary, so many aspects affect the dynamic balance of ABA in plants that further investigating the regulatory mechanism and identifying new components responsive to ABA homoeostasis will provide important theoretical bases for genetic breeding.
